# TMEM106C contributes to the malignant characteristics and poor prognosis of hepatocellular carcinoma

**DOI:** 10.18632/aging.202487

**Published:** 2021-02-11

**Authors:** Jicheng Duan, Youwen Qian, Xiaohui Fu, Meiling Chen, Kai Liu, Hu Liu, Jiahe Yang, Chen Liu, Yanxin Chang

**Affiliations:** 1 Biliary Tract Surgery Department, Eastern Hepatobiliary Surgery Hospital, Second Military Medical University, Shanghai 200438, PR China; 2 Department of Pathology, Eastern Hepatobiliary Surgery Hospital, Second Military Medical University, Shanghai 200438, PR China; 3 Department of Anesthesiology, Shanghai Yangpu District Central Hospital, Shanghai Tongji University, Shanghai 200090, PR China

**Keywords:** hepatocellular carcinoma, TMEM106C, metastasis, proliferation, bioinformatics

## Abstract

Transmembrane protein (TMEM) is a kind of integral membrane protein that spans biological membranes. The functions of most members of the TMEM family are unknown. Here, we conducted bioinformatic analysis and biological validation to investigate the role of TMEM106C in HCC. First, GEPIA and Oncomine^TM^ were used to analyze TMEM106C expression, which was verified by real-time PCR and western blot analyses. Then, the biological functions of TMEM106C were explored by CCK8 and transwell assays. The prognostic value of TMEM106C was analyzed by UALCAN. LinkedOmics was used to analyze TMEM106C pathways generated by Gene Ontology. A protein-protein interaction network (PPI) was constructed by GeneMANIA. We demonstrated that TMEM106C was overexpressed in HCC and that inhibition of TMEM106C significantly suppressed the proliferation and metastasis of HCC through targeting CENPM and DLC-1. Upregulation of TMEM106C was closely correlated with sex, tumor stage, tumor grade and prognosis. Overexpression of TMEM106C was linked to functional networks involving organelle fission and cell cycle signaling pathways through the regulation of CDK kinases, E2F1 transcription factors and miRNAs. Our data demonstrated that TMEM106C contributes to malignant characteristics and poor prognosis in HCC, which may serve as a prognostic biomarker and potential therapeutic target.

## INTRODUCTION

Hepatocellular carcinoma (HCC) is the most common primary liver cancer and is the sixth most frequent kind of tumor [1, 2]. It is the second leading cause of cancer-related death worldwide due to its high morbidity and mortality [2]. Liver resection, transplantation and radiofrequency ablation (RFA) therapy are potential curative treatments for HCC. However, long-term outcomes of HCC patients are high rates of recurrence or metastasis [3]. It is well known that intricate genetic or epigenetic alterations are involved in HCC progression [4]. Many studies have explored the molecules and pathways governing HCC progression, but the outcomes of patients with HCC remain unsatisfactory [5, 6]. Therefore, exploring novel molecular mechanisms and identifying valuable markers of HCC are extremely urgent.

TMEM proteins span the entire width of the lipid bilayer and are often considered to be components of various cell membranes, such as mitochondrial, endoplasmic reticulum, lysosome and Golgi membranes [7]. Many TMEM proteins function as biological channels that permit the transport of some substances across the cell membranes. However, their biological functions remain to be elucidated. Under both physiological or pathological conditions, TMEM proteins play pivotal roles in diverse biological processes, such as epidermal keratinization, autophagy, smooth muscle contraction, protein glycosylation, liver development and differentiation, and regulation of the immune response [8–12]. Despite the different roles or localizations of TMEM proteins, many of them are implicated in cancers, including lymphomas, colorectal cancer, hepatic cancer, and lung cancer [13–16]. Furthermore, there are increasing evidences that TMEM proteins can function as tumor suppressors as well as oncogenes. In many cancers, aberrant expression of TMEM proteins has already been reported to serve as a new prognostic biomarker and treatment target for cancer patients [17–19]. A further characterization of such proteins could help to better understand their roles in cancers, and some could be new therapeutic targets.

It was reported that TMEM45A is involved in the chemoresistance of liver cancers [20]. However, roles of other TMEM proteins in HCC are still unknown. Here, we studied TMEM106C expression in patients with HCC using data from The Cancer Genome Atlas (TCGA) and various other public databases. Biological validation was conducted to verify TMEM106C expression and to explore the functions and target genes in HCC cells. Moreover, we investigated the significance and prognostic value and analyzed functional networks related to TMEM106C in HCC using multiple analyses in addition to biological validation; these data may provide new clues for potential therapeutic targets and strategies against HCC.

## RESULTS

### TMEM106C expression in HCC

**Figure 1:**
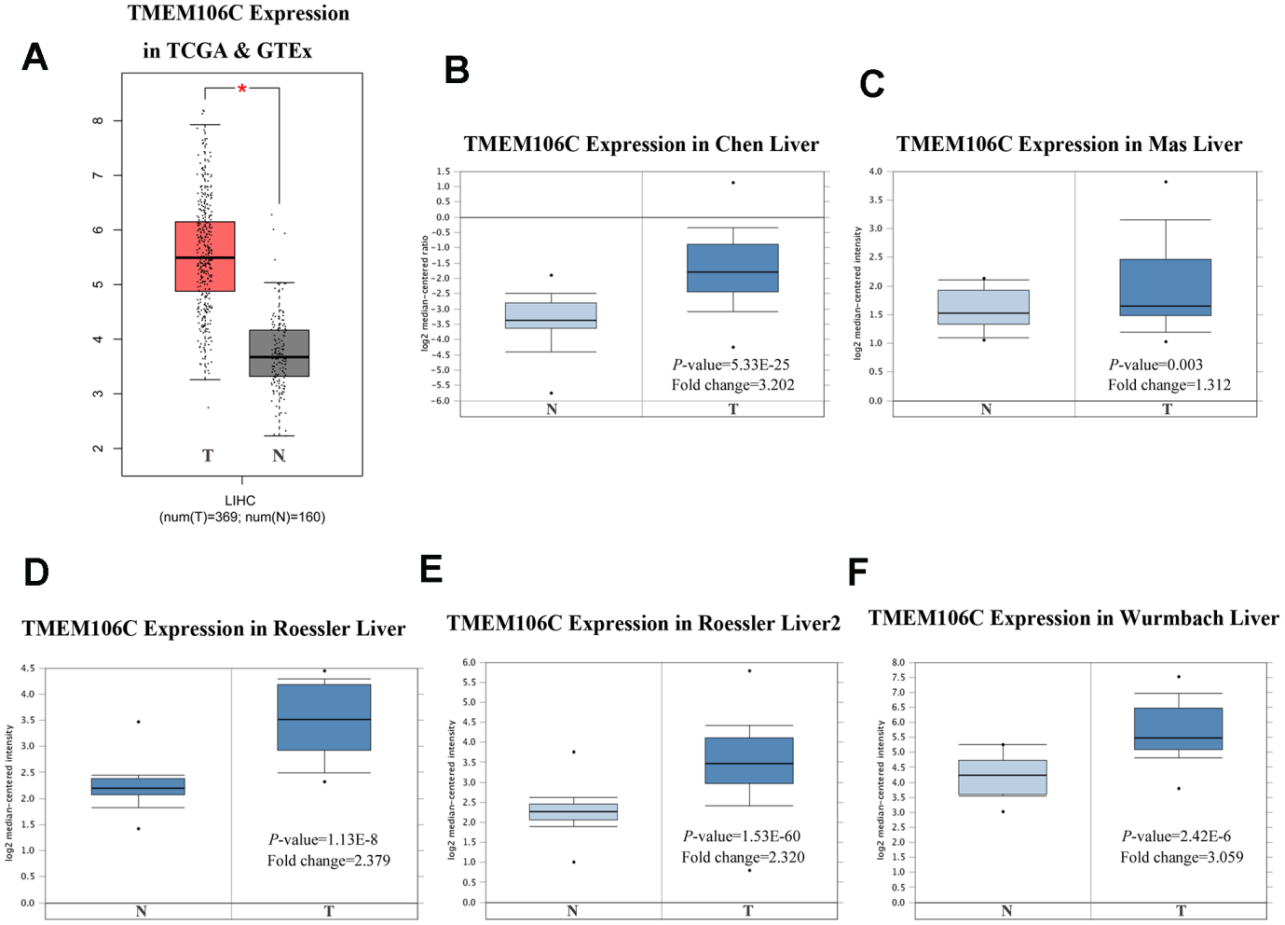
**TMEM106C transcription in tumor tissues HCC (GEPIA and Oncomine).** (**A**) Boxplot showing TMEM106C mRNA levels in TCGA and GTEx from GEPIA. *P < 0.05. (**B**–**F**) Boxplot showing TMEM106C mRNA levels in Chen Liver, MasLiver, RoesslerLiver, RoesslerLiver2, and Wurmbach Liver datasets from Oncomine. (T=tumor, N=normal).

**Figure 2:**
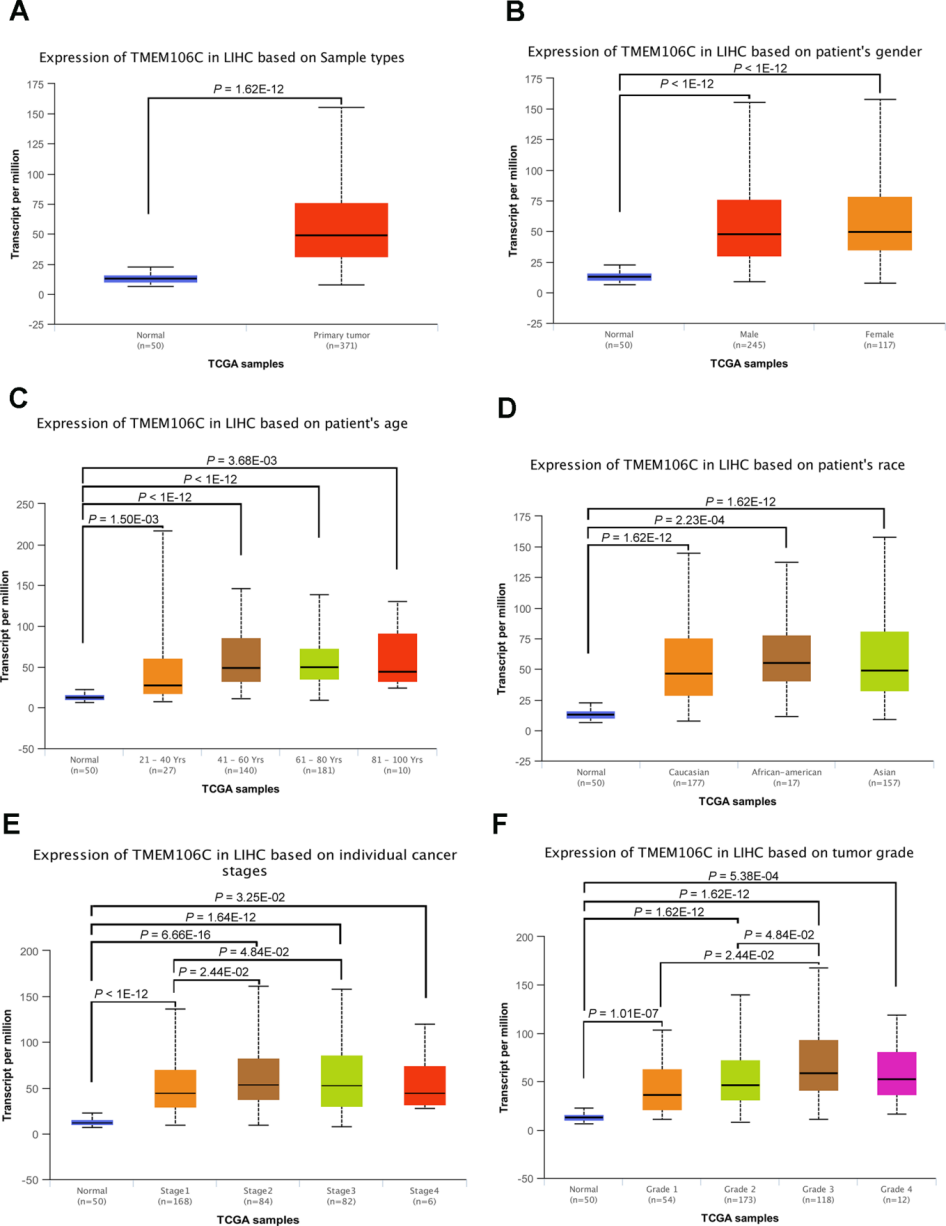
**TMEM106C transcription in subgroups of HCC patients, stratified by gender, age, race, tumor stage and tumor grade (UALCAN).** (**A**) Relative level of TMEM106C in normal liver and LIHC samples. (**B**) Boxplot showing the relative expression of TMEM106C in healthy controls and male or female LIHC patients. (**C**) Relative level of TMEM106C in healthy controls of any age and LIHC patients of different age periods. (**D**) Relative level of TMEM106C in healthy controls of any ethnicity and LIHC patients of Caucasian, African-American or Asian ethnicity. (**E**) Boxplot showing the relative expression of TEM106C in healthy controls and LIHC patients in different stages. (**F**) Relative level of TMEM106C in healthy controls and LIHC patients with grades 1, 2, 3 or 4. 31 Data are shown as the mean ± SE.

GEPIA (Gene Expression Profiling Interactive Analysis) is a web-based tool used to deliver fast and customizable functionalities based on TCGA and GTEx data, and it provides key interactive and customizable functions, including differential expression analysis, profiling plotting, correlation analysis, patient survival analysis, similar gene detection and dimensionality reduction analysis [21]. Based on the GEPIA database, we initially discovered that TMEM106C is highly expressed in HCC tumor tissues compared to normal liver tissues (Figure 1A, *P* < 0.01). Then, we further evaluated TMEM106C transcription levels in multiple HCC studies from TCGA and the Gene Expression Omnibus (GEO). Data from the Oncomine 4.5 database revealed that the TMEM106C expression level is consistently significantly higher in HCC tissues than it is in normal tissues, as seen in the following studies: Chen Liver, Mas liver, Roessler liver, Roessler liver2 and Wurmbach Liver (Figure 1B–1F, *P* < 0.01). Furthermore, the transcription level of TMEM106C in HCC was significantly higher in the subgroup analysis based on gender, age, ethnicity, disease stage and tumor grade (Figure 2), as revealed in 371 liver hepatocellular carcinoma (LIHC) samples in TCGA. As shown in Figure 2E, 2F, the higher transcription level of TMEM106C was almost parallel to that of tumor stage and tumor grade, indicating that TMEM106C is closely related to the malignant biological behavior of HCC. However, the TMEM106C transcripts of stage 4 and grade 4 were not significantly different from those of the other stages, which may be due to the small sample size or the influence of tumor microenvironment. Because increasing lines of evidence show that the biological behavior of cancer is not exclusively attributable to cancer cells themselves but also radically influenced by the microenvironment [22, 23]. After distant metastasis, the biological behavior of cancer cells of stage 4 in the different microenvironment may be different from before.

**Figure 3:**
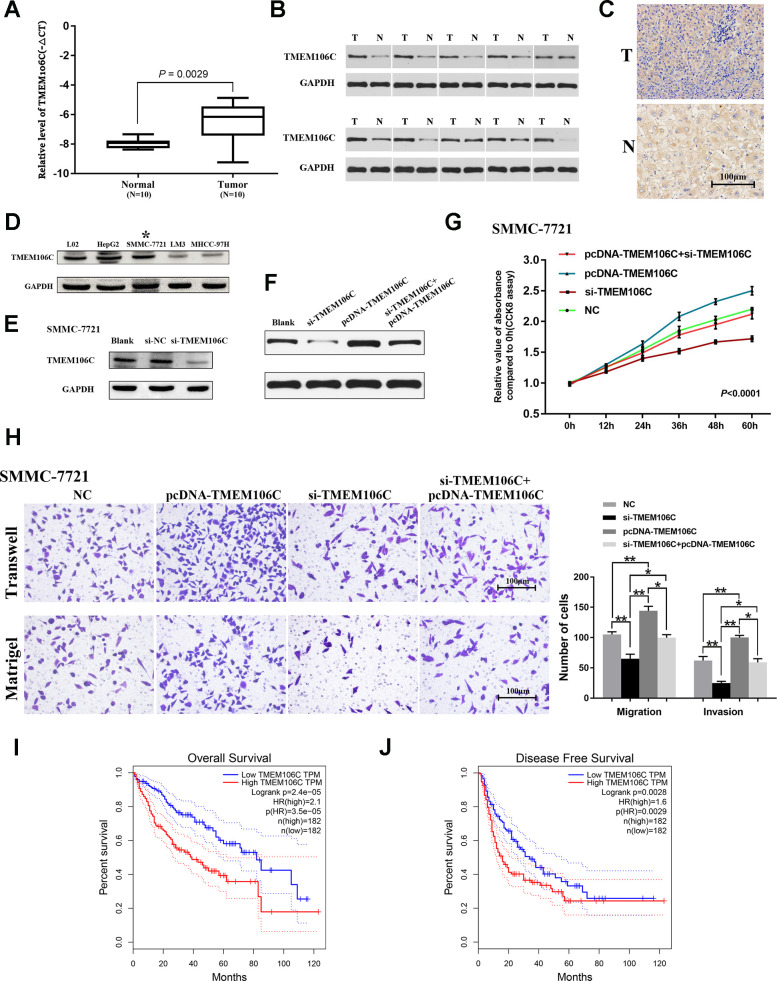
**Expression validation, functional exploration and prognostic value of TMEM106C in HCC.** (**A**) Relative expression level of TMEM106C in 10 pairs of HCC samples (tumor tissues and adjacent normal liver tissues), as assessed by real-time PCR. (**B**) The protein expression level of TMEM106C in 10 pairs of HCC samples, as assessed by western blot. (**C**) TMEM106C IHC staining in HCC and adjacent normal liver tissue. 400×. (**D**) The protein expression level of TMEM106C in the normal liver cell line L02 and in different HCC cell lines. The cell line SMMC-7721 was selected for further study. (**E**) siRNAs targeting TMEM106C were transfected into SMMC-7721 cells for 48 h, and then all cell lysates were harvested for western blotting. (**F**) TMEM106C levels under si-TMEM106C, pcDNA-TMEM106C plasmid or si-TMEM106C plus pcDNA-TMEM106C plasmid assessed by western blot. (**G**) si-TMEM106C (50 nM) and pcDNA-TMEM106C plasmid plus si-TMEM106C were transfected into SMMC-7721 cells and were compared to untransfected control cells. Every 12 h, cell numbers were measured by CCK8 assay. NC represents pcDNA3.1, si-NC, and blank control, which were proven to not be different from each other. *P* < 0.01. 32 (**H**) Transwell migration and invasion assays of SMMC-7721 cells after transient transfection with si-TMEM106C or pcDNA-TMEM106C plasmid plus si-TMEM106C or not. The migration and invasion cell numbers are shown in histograms (mean ± SD). NC represents pcDNA3.1, si-NC, and blank control, which were proven to not be different from each other. 400×. *P < 0.05, **P < 0.01. (**I**) The overall survival rates of 364 HCC patients were compared between the TMEM106C high and low expression groups using Kaplan-Meier analysis (GEPIA). (**J**) The disease-free survival rates of 364 HCC patients were compared between the TMEM106C high and low expression groups using Kaplan-Meier analysis (GEPIA).

To further validate the level of TMEM106C in HCC, real-time PCR was performed in 10 pairs of fresh human HCC tissues. Compared with their noncancerous counterparts, the levels of TMEM106C were much higher in cancer tissues (Figure 3A, Supplementary Figure 1A, 1B). Western blot and immunohistochemistry data also revealed that TMEM106C is highly expressed in HCC (Figure 3B, 3C). Preliminarily, it was speculated that TMEM106C might act as a potential diagnostic indicator or oncogene in HCC.

### The biological functions of TMEM106C in HCC

To further elucidate the underlying biological function of TMEM106C in HCC, several HCC cell lines, HepG2, SMMC-7721, LM3, MHCC-97H and normal liver cell line L02, were employed. The expression level of TMEM106C was detected. We found that TMEM106C is highly expressed both in HepG2 and SMMC-7721 cell lines, as well as in L02 cells (Figure 3D). Considering that the transfection efficiency of the HepG2 cell line is low, SMMC-7721 cells were selected for further study. We constructed a pcDNA-TMEM106C plasmid that could obviously upregulate the expression of TMEM106C (Supplementary Figure 1A), and we also synthesized siRNA that could effectively decrease the expression level of TMEM106C in SMMC-7721 cells (Figure 3E, Supplementary Figure 1C). Then, a CCK8 assay was performed. Overexpression of TMEM106C clearly promoted the proliferation rate of SMMC-7721 cells, while inhibition of TMEM106C expression by si-TMEM106C significantly suppressed the proliferation rate of HCC cells compared with that of the NC group (Figure 3F, 3G, *P* < 0.01). Furthermore, as illustrated by the transwell assay with or without Matrigel, overexpression of TMEM106C obviously promoted the migration and invasion ability of cancer cells compared with the NC group, while low expression of TMEM106C impaired this effect (Figure 3H, **P < 0.01). Collectively, our data demonstrated that TMEM106C plays a crucial role in promoting both cell proliferation and metastasis *in vitro*, which needs further validation *in vivo* in the future.

### The prognostic value of TMEM106C in HCC

We next wanted to determine the impact of elevated TMEM106C on clinical HCC patients. Therefore, the GEPIA database was employed to clarify the prognostic value of TMEM106C. As Figure 3I shows, patients that exhibited high TMEM106C expression had a much worse overall survival (OS) time than patients with low expression (*P* < 0.0001, median OS time were 39 and 82 months, respectively). Additionally, elevated TMEM106C expression was associated with poor disease-free survival (DFS) time (*P* < 0.01, median DFS time were 17 and 34 months, respectively) (Figure 3J). These data suggest that TMEM106C could be used as a prognostic marker of HCC, and more importantly, they show that inhibition of TMEM106C may improve the poor prognosis of HCC.

### Target gene analysis

**Figure 4:**
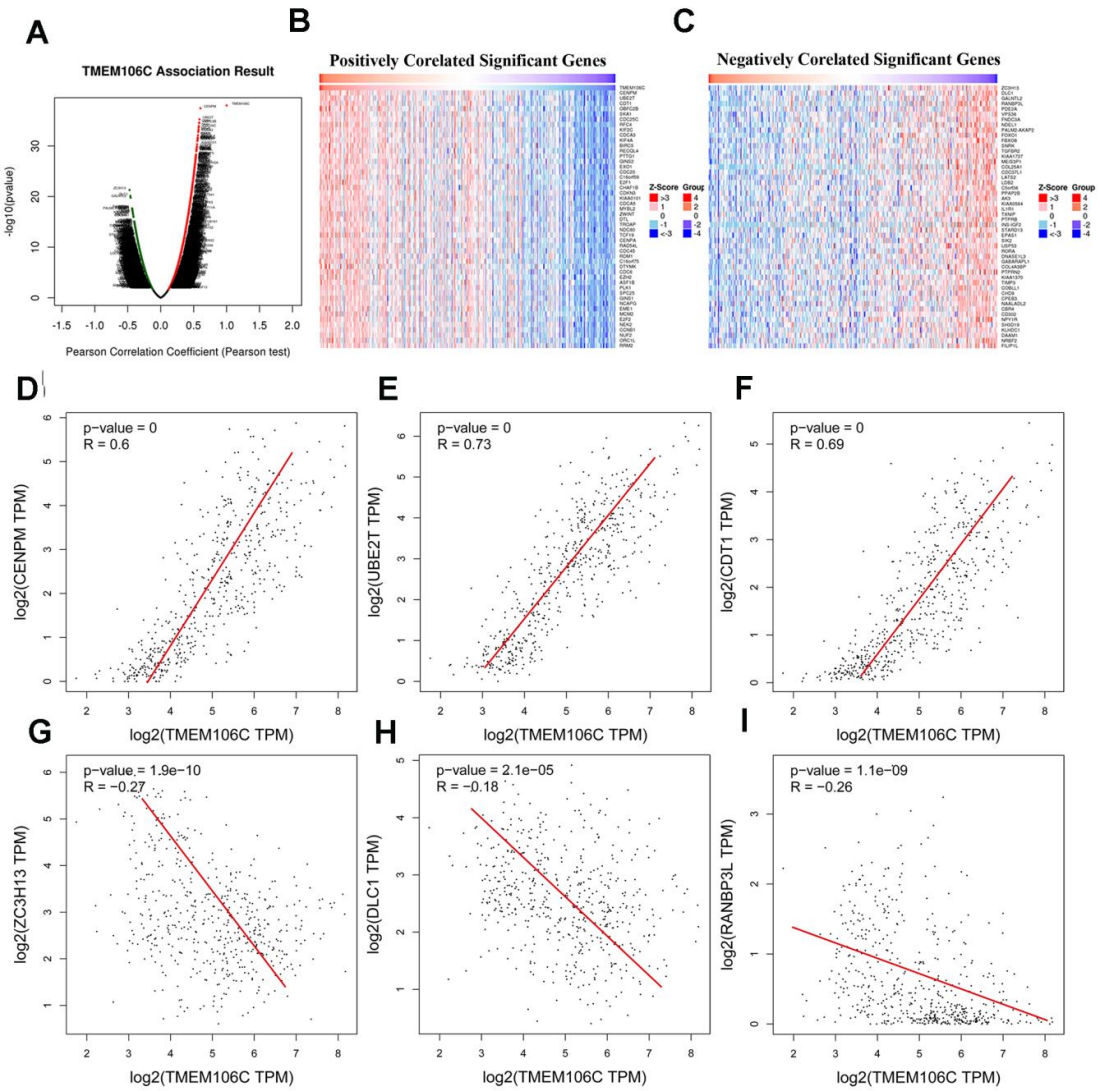
**TMEM106C expression correlated genes in HCC (LinkedOmics and GEPIA).** (**A**) Correlations between TMEM106C and differentially expressed genes in LIHC from LinkedOmics (Pearson test). Red indicates positively correlated genes, and green indicates negatively correlated genes. (**B**, **C**) Heat maps showing the top 50 genes positively and negatively correlated with TMEM106C in LIHC. (**D**–**F**) The scatter plots show the Pearson correlation of TMEM106C expression with the most positively correlated genes: CENPM, UBE2T and CDT1 (GEPIA). (**G**–**I**) The scatter plot shows the Pearson correlation of TMEM106C expression with the most 33 negatively correlated genes: ZC3H13, DLC1 and RANBP3L (GEPIA).

To better understand the underlying molecular mechanisms by which TMEM106C regulates the biological characteristics of HCC, the LinkFinder module of LinkedOmics was employed to analyze differentially expressed genes that are closely related to TMEM106C as determined by TCGA mRNA sequencing data from 371 LIHC patients. In total, 3213 genes (red dots) were identified as being positively correlated with TMEM106, while 3010 genes (green dots) showed negative correlation (Figure 4A, FDR < 0.01; Supplementary Table 1). Additionally, the top 50 genes that were most significantly positively and negatively correlated with TMEM106C are shown on a heat map (Figure 4B, 4C). Using gene correlation analysis of GEPIA, TMEM106C expression was revealed to have a significant positive correlation with the expression of CENPM (centromere protein M) [24] (Pearson correlation = 0.6, *P* = 3.62E-38), UBE2T (ubiquitin conjugating enzyme E2 T) [25] (Pearson correlation = 0.73, *P* = 5.56E-36), and CDT1 (chromatin licensing and DNA replication factor 1) [26] (Pearson correlation = 0.69, *P* = 2.41E-35) (Figure 4E–4G), which regulate chromosome segregation during cell division, protein ubiquitination and DNA damage, and DNA replication licensing, respectively. In particular, the aberrant expression of CDT1 is closely linked to DNA replication defects, aneuploidy and genomic instability, which are considered to be integral to precancerous states and essential elements for malignant transformation. Furthermore, CDT1 expression is downregulated in human tumor specimens, so it may represent a novel marker useful for cancer diagnosis and prognosis [26]. Meanwhile, TMEM106C was also found to be negatively associated with ZC3H13 (zinc finger CCCH-type containing 13) [27] (Pearson correlation = -2.7, *P* = 1.9E-10), DLC1 (deleted in liver cancer-1) [28, 29] (Pearson correlation = -1.8, *P* = 2.1E-05), and RANBP3L (RAN binding protein 3 like) [30] (Pearson correlation = -2.60, *P* = 1.1E-9) (Figure 4H–4J), which have been reported to mediate RNA N6-methyladenosine (m6A) methylation, suppress cancer metastasis, terminate BMP signaling and regulate mesenchymal stem cell differentiation, respectively. These data indicated that TMEM106C has an extensive impact on the other related genes, and TMEM106C serves as an oncogene in HCC that may depend on interacting with such targets.

### Verification of potential target genes

**Figure 5:**
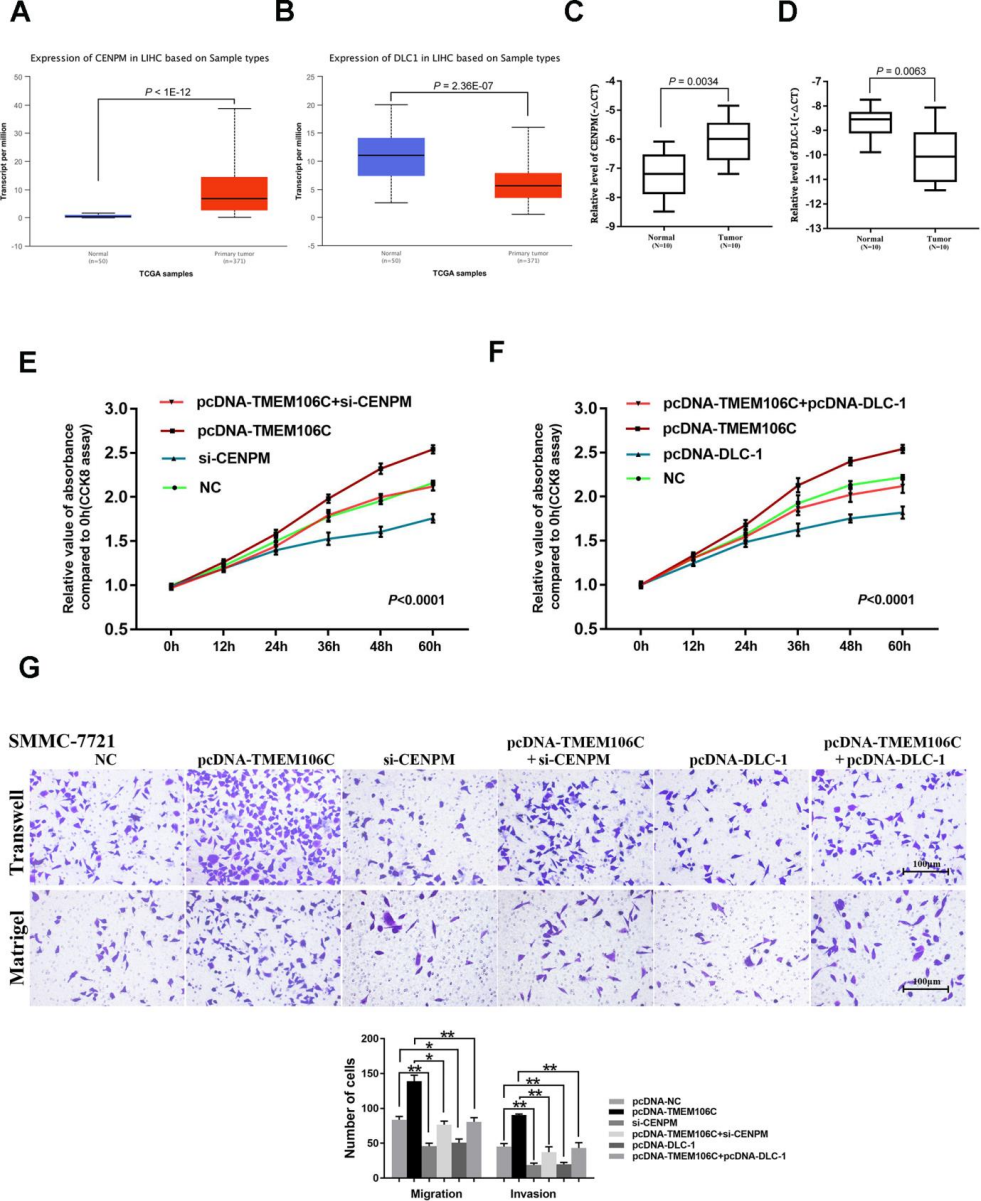
**The verification of TMEM106C potentially related target genes in HCC.** (**A**) Relative level of CENPM in normal liver and LIHC samples (UALCAN). (**B**) Relative level of DLC-1 in normal liver and LIHC samples (UALCAN). (**C**) Relative expression level of CENPM in 10 pairs of HCC samples (tumor tissues and adjacent normal liver tissues), as assessed by real-time PCR. (**D**) Relative expression level of DLC-1 in 10 pairs of HCC samples (tumor tissues and adjacent normal liver tissues), as assessed by real-time PCR. (**E**) si-CENPM (50 nM) and pcDNA-TMEM106C plasmid plus si-CENPM were transfected into SMMC-7721 cells. Every 12h, the cell number was measured by CCK8 assay. NC represents pcDNA3.1, si-NC, and blank control, which were proven to not be different from each other. 400×. *P* < 0.001. (**F**) pcDNA-TMEM06C and pcDNA-DLC-1 plus pcDNA-TMEM06C plasmid (or no plasmid control) were transfected into SMMC-7721 cells. Every 12 h, the cell number was measured using the CCK8 assay. NC represents pcDNA3.1 and the blank control, which was proven to not be different from each other. *P* < 0.001. (**G**) Transwell migration and invasion assays of SMMC-7721 cells after transient transfection with pcDNA-TMEM06C, si-CENPM, pcDNA-TMEM06C and si-CENPM, pcDNA-DLC-1 plus 34 pcDNA-TMEM06C plasmid (or no plasmid control). The migration and invasion cell numbers are shown in histograms (mean ± SD). NC represents pcDNA3.1, si-NC, and blank control, which were proven to not be different from each other. *P < 0.05, **P < 0.01.

As a gene with expression that is positively related to that of TMEM106C, CENPM has been documented as an oncogene in HCC [24]. Additionally, the negatively correlated gene DLC-1 was found to be a metastasis suppressor gene in various tumors [28, 29]. To address the mechanisms by which TMEM106C functions in HCC, we further explored CENPM and DLC-1 transcription levels by UALCAN. CENPM was highly expressed in tumor tissues, while DLC-1 was significantly downregulated in HCC (Figure 5A, 5B). The results were verified by real-time PCR in 10 pairs of HCC tissues (Figure 5C, 5D). Furthermore, silencing CENPM by si-CENPM significantly suppressed the proliferation, migration and invasion ability of HCC cells, which could also attenuate the effects induced by TMEM106C (Figure 5E, 5G). Moreover, overexpression of DLC-1 by transient transfection with a pcDNA-DLC-1 plasmid suppressed the proliferation-promoting effect and migration/invasion-promoting effect of TMEM106C upregulation (Figure 5F, 5G). These results indicated that CENPM and DLC-1 may be positive and negative target genes of TMEM106C, respectively, and that TMEM106C may play key roles in HCC through manipulating them.

### Functional and pathway enrichment analysis

**Figure 6:**
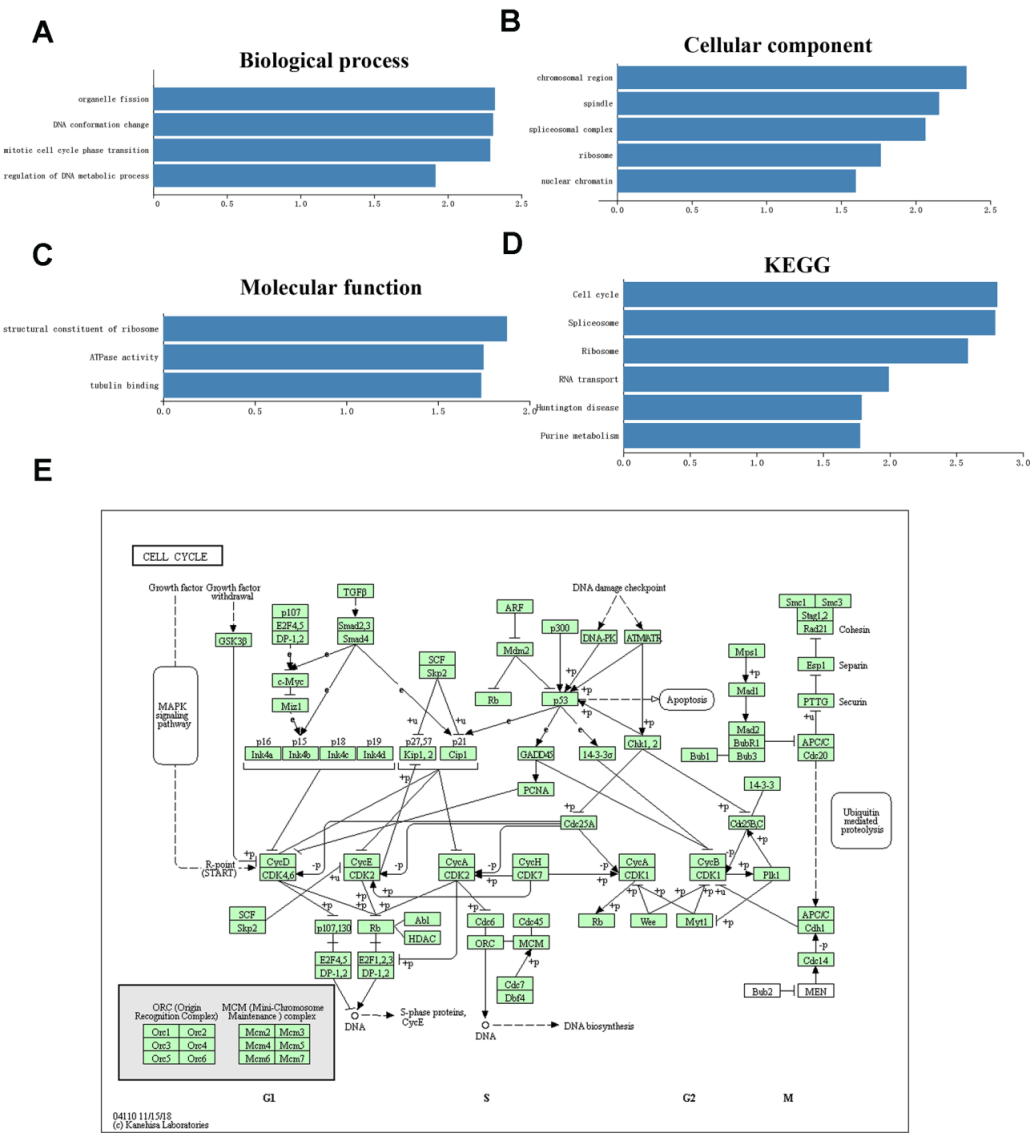
**Significantly enriched GO annotations and KEGG pathways of TMEM106C in HCC analyzed by GSEA (LinkedOmics).** (**A**) Cellular components. (**B**) Biological processes. (**C**) Molecular functions. (**D**) KEGG pathway analysis. FDR=0. (**E**) KEGG pathway annotations of the cell cycle pathway.

We also performed GO classification and KEGG pathway enrichment analysis on TMEM106-related differentially expressed genes. We found some significant GO terms, including organelle fission, DNA conformation, mitotic cell cycle phase transition, tubulin binding and others that are mainly involved in the regulation of cell proliferation, cell cycle process and cell adhesion (Figure 6A–6C). Additionally, KEGG pathway analysis showed that TMEM106C-related genes were enriched in the cell cycle, splicesome, ribosome and RNA transport pathways (Figure 6D, 6E). Taken together, these results suggest that TMEM106C plays a significant role in the regulation of cellular proliferation and cancer cell motility.

### TMEM106C protein-protein interaction (PPI) network construction

**Figure 7:**
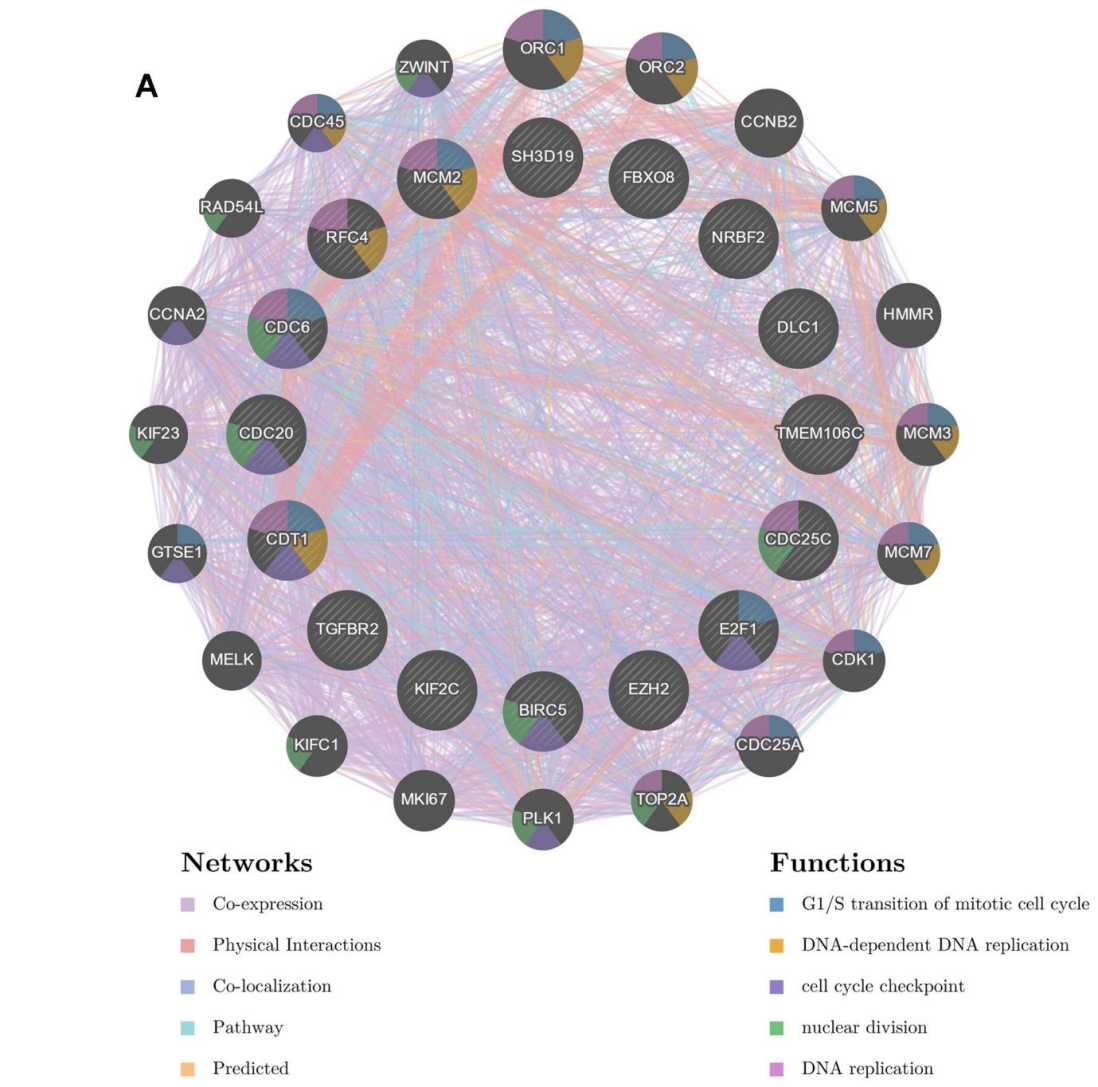
**Interaction network of associated genes generated by GENEMANIA.** (**A**) The PPI network and functional analysis consisted of the TF-kinase-miRNA-TMME106C interaction. These genes were linked by different colors indicating the following relation: coexpression, physical interaction, colocalization, pathway or predicted, while the different colors for the network nodes indicate the biological functions of the set of enriched genes.

To further investigate targets of TMEM106C in HCC, TMEM106C-related kinase targets, transcription factor targets and miRNA target networks of positively or negatively correlated gene sets were analyzed by the GSEA module of LinkedOmics. The kinase-target network was closely associated with cyclin-dependent kinase 1 (CDK1), polo-like kinase 1 (PLK1), Rad3-related (ATR), cyclin-dependent kinase 2 (CDK2) and aurora kinase B (AURKB). The transcription factor-target network was mainly related to the E2F transcription factor 1 (E2F1) family, as well as nuclear respiratory factor 1 (NRF1) and ETS transcription factor 1 (ELK1). Meanwhile, the miRNA-target network was mostly associated with the TTTGCAC-binding family, miR-19A/19B (Table 1). We selected the top 5 most related genes out of the CDK1 kinase, E2F1 transcription factor and miRNA-19A/19B groups. Next, we used GeneMANIA to generate a PPI network to reveal correlations among genes from the CDK1 kinase, E2F1 transcription factor and miRNA-19A/19B groups. The network consists of 36 genes, including 15 selected proteins, as well as TMEM106C and 20 additional proteins that were identified by GeneMANIA (Figure 7A). There are five types of interrelationships: coexpression (75.33%), physical interactions (10.89%), colocalization (5.92%), pathway (5.92%) and predicted (3.00%), all of which could be found in published papers. Functional annotation analysis demonstrated that this subnetwork is mainly involved in regulation of the G1/S transition during mitosis, DNA-dependent DNA replication and the cell cycle checkpoint. Based on the signal pathway analysis, PPI analysis and target gene validation results, further study on the potential signal pathways by which TMEM106C functions in HCC is needed.

**Table 1:** The kinase, transcription factor-target and miRNA networks of TMEM106C in HCC (LinkedOmics).

**Enriched category**	**Geneset**	**Leading edge number**	**NES**	**P**	**FDR**
**Kinase Target**	Kinase_CDK1	89	2.3165	0	0
	Kinase_PLK1	30	2.3698	0	0
	Kinase_ATR	26	2.0808	0	0
	Kinase_CDK2	90	2.0705	0	0
**Transcription Factor** **Target** **miRNA Target**	Kinase_AURKB V$E2F1_Q6 RCGCANGCGY_V$NRF1_Q6 SCGGAAGY_V$ELK1_02 **TTTGCAC**,MIR-19A,MIR-19B	25 80 192 298 190	1.9999 2.2781 1.4949 1.4758 -2.0434	0 0 0 0 0	0 0 0.027832 0.040879 0
	**GCACTTT**,MIR-17-5P,MIR-20A,MIR-106A,MIR-106B,MIR-20B,MIR-519D **TGAATGT**,MIR-181A,MIR-181B,MIR-181C,MIR-181D **CAGTATT**,MIR-200B,MIR-200C,MIR-429 **CTTTGTA**,MIR-524	177 160 142 130	-1.9865 -1.9959 -1.8938 -1.9302	0 0 0 0	0 0 0 0

Abbreviations: NES, Normalized Enrichment Score; FDR, false discovery rate from Benjamini and Hochberg from gene set enrichment analysis (GSEA); V$, the annotation found in Molecular Signatures Database (MSigDB) for transcription factors (TF).

## DISCUSSION

HCC represents one of the few cancers for which locoregional treatments are recognized as being able to cure or prolong survival, which is due to the unique fact that most HCC cases occur in patients with underlying virus- or alcohol-related cirrhosis. Generally, HCC patients may undergo surgical resection, tumor ablation, or liver transplantation as potentially curative therapies. However, only 5%-10% of patients with HCC are eligible for hepatectomy, since a majority of patients present with advanced stage disease. Additionally, both liver transplantation and tumor ablation have limitations or strict indications. To date, the diagnosis of HCC is made mainly based on findings from biopsy or imaging analyses. Molecular markers are still not used in the diagnosis or determination of prognosis and treatment for patients. The long-term outcomes remain unsatisfactory for HCC patients due to the high rates of recurrence or metastasis even if they are treated with the multikinase inhibitors sorafenib, egorafenib or lenvatinib [31]. The challenges are mainly centered around how to screen HCC patients at an earlier stage and thereby conduct timely curative treatment. Thus, exploring the molecular mechanisms and identifying valuable markers of HCC are urgently needed.

The TMEM protein family plays critical roles in regulating numerous physiological processes, including neuronal excitability, smooth muscle contraction, nociception plasma membrane ion channel formation, signal transduction, cellular chemotaxis, adhesion, apoptosis, and autophagy [8, 17, 32–39]. Additionally, emerging evidence reveals that the TMEM protein family is significantly correlated with malignant progression and chemotherapeutic resistance in various cancers, even though the functions of most TMEM proteins remain unknown and warrant detailed investigation [40–44]. To gain more detailed insights into the potential functions of TMEM106C in HCC and its regulatory network, we performed bioinformatic analysis in addition to biological validation to guide future research in HCC. The data suggested that TMEM106C is highly expressed in HCC and deserves further clinical analysis as a potential diagnostic and prognostic marker.

To date, high-throughput RNA sequencing (RNA-Seq) has emerged as a powerful method for transcriptomic analysis, and a tremendous amount of RNA sequencing data have been produced by large consortium projects such as TCGA and GTEx. However, professional or complicated bioinformatic analysis from such a database mainly depends on R, PHP or Python scripts, which is a challenge for many researchers. Fortunately, increasing the number of data servers based on TCGA, GTEx or others facilitates data mining and analysis. This study used online tools to perform target gene analysis on tumor data from public databases. Compared with traditional chip screening, this method has the advantages of being low cost, having a larger sample size and being simple. For example, GEPIA is an interactive web application for gene expression analysis based on 9736 tumors and 8587 normal samples from the TCGA and GTEx databases. Such methodology enables experimental biologists without any computational programming skills to perform a diverse range of gene expression analyses, profiling plotting, correlation analysis, patient survival analysis, similar gene detection and dimensionality reduction analysis [23]. The LinkedOmics database is a web tool containing multiomics data and clinical data for 32 cancer types from TCGA project. It has a very low barrier to conduct within- and across-omics association, pathway analysis, multiomics and pancancer analysis [45]. GeneMANIA is a flexible user-friendly website for PPI construction based on genomic, proteomic, and gene function data. We can use such interactive and friendly web tools easily and professionally for data mining, deeper understanding of gene functions and biological patterns, finding candidate drug targets and identifying biomarkers for disease classification and diagnosis [46].

Gene expression is regulated in almost two ways: regulation of transcription from DNA to RNA by TFs, regulation of RNA stability by miRNAs, and activation by kinases or other genes. In this study, we first analyzed the differential gene expression of TMEM106C in HCC samples and normal liver samples and investigated the functions and pathways with which differentially expressed genes were mainly associated. We also constructed a TF-kinase-miRNA-gene regulatory network based on closely related mRNA and miRNA expression data to better clarify the cellular mechanisms of HCC. Here, we found that some crucial gene sets, such as kinase CDK1, TF E2F1, and miR-19A/19B, are massively altered in HCC. These genes and miRNAs can potentially be applied not only as novel biomarkers but also as therapeutic targets. For example, the positively related genes CENPM and UBE2T and the negatively correlated genes ZH3H13 and DLC1. CENMP was identified as a key candidate gene involved in melanoma metastasis [47], and low expression of CENPM correlated with better progression-free survival in bladder cancer than high expression [48]. UBE2T silencing suppresses proliferation and induces cell cycle arrest and apoptosis in bladder cancer cells [49], and UBE2T knockdown inhibits gastric cancer progression [50]. Meanwhile, as an emerging metastasis suppressor gene, DLC1 can inhibit cancer progression and oncogenic autophagy in HCC [28, 51]. Based on the background biological information, we demonstrated that TMEM106C promoted proliferation and metastasis through the regulation of CENPM and DLC-1. This evidence confirms that TMEM106C-related genes may be the downstream of TMEM106C. CDK1 and miR-19A/19B were located in the central hub of our TF-kinase-miRNA-gene network. CDK1 plays a key role in the control of the eukaryotic cell cycle by modulating the centrosome cycle as well as mitotic onset, and deregulation of CDK1 is considered a promising future cancer treatment [52, 53]. Furthermore, overexpression of miR-19A inhibits colorectal cancer angiogenesis by suppressing KRAS expression [54]. Taken together, these selected critical related genes, kinases, transcription factors and miRNAs may be the hub genes that play crucial roles in the TMEM106C-related network in HCC development, which also suggest the need for further validation in clarifying more thoroughly the mechanism of TMEM106C.

Generally, all bioinformatic analyses have some amount of limitations. For example, the clinical data are not all detailed enough, and the data contain ethnic differences. Most importantly, the results of the analysis need to be verified with clinical samples. Therefore, in our study, we conducted experiments using fresh HCC tumor tissues and HCC cell lines to confirm the expression level of TMEM106C. Moreover, we explored the exact biological functions of TMEM106C *in vitro*. The validation data make our study more convincing and important. However, there are still several limitations of this study that warrant discussion. First, the clinical data from TCGA are not complete enough to conduct multivariate analysis and clarify whether TMEM106C is an independent prognostic factor in HCC patients. Second, the biological data need further experiments *in vivo* in the future. Third, the network of TMEM106C in HCC is derived from RNA sequencing data and existing studies of HCC or other kinds of cancers; hence, further validation is still needed to authenticate the potential mechanisms of TMEM106C in HCC.

Collectively, our study provides multiple analyses that show the importance of TMEM106C in HCC development and its potential as a marker in HCC. We discovered that TMEM106C is highly expressed in HCC, and biological validation demonstrated that the overexpression of TMEM106C may contribute to the proliferation and metastasis of HCC by targeting CENPM and DLC-1. Furthermore, the elevated level of TMEM106C is associated with poor prognosis of HCC. Overall, our findings will improve our understanding of the molecular mechanisms of HCC and aid in finding potential targets for future diagnostic and therapeutic purposes.

## MATERIALS AND METHODS

### Collection of human tissue specimens

Ten pairs of tumor and adjacent normal tissues were obtained from patients with HCC after hepatectomy in Eastern Hepatobiliary Surgery Hospital (Shanghai, China). All specimens were confirmed by the Department of Pathology after curative resection, and they were kept in a liquid nitrogen canister before use. All human sample collection procedures were approved by the Research Ethics committee. Written informed consent was obtained from all participants.

### Cell culture and transfection

HCC cell lines (HepG2, SMMC-7721, LM3, and MHCC-97H), as well as a normal human liver cell line, L02, were obtained from the Cell Resource Center of the Chinese Academy of Sciences (Shanghai, China). Cells were routinely cultured in DMEM (Gibco, USA) supplemented with 10% fetal bovine serum (FBS; PAA Laboratories, Pasching, Austria) and were maintained in a 37° C humidified incubator containing a 5% CO_2_ atmosphere.

TMEM106C and DLC-1 expression plasmids were constructed using the pcDNA3.1 expression vector. The coding sequences of TMEM106C and DLC-1 (Supplementary Table 3) were synthetized by ELK biotechnology (Wuhan, China). A TMEM106C CRISPR/Cas9 KO plasmid was purchased from Santa Cruz Biotechnology (CA, USA). Small interfering RNAs (siRNAs) against TMEM106C (si-TMEM106C) and CENPM (si-CENPM) (Supplementary Table 4) as well as a nontargeting control (si-NC) were purchased from RiboBio (Guangzhou, China). Transfections were performed using Lipofectamine 2000 (Invitrogen Carlsbad, CA, USA) according to the manufacturer’s protocols.

### RNA extraction and quantitative real-time PCR

TRIzol (Takara, Dalian, China) was used to extract total RNA from tissues. Total RNA was reverse transcribed to generate cDNA. TMEM106C mRNA levels were examined using a SYBR Green-based real-time PCR kit (Vazyme Biotech, Nanjing, China) on an ABI 7300 Platform (Applied Biosystems, CA). Data were analyzed based on the comparative cycle threshold (Ct) values. The primer sequences of TMEM106C, GAPDH, CENPM, and DLC-1 can be found in Supplementary Table 2.

### Cell proliferation

Cell proliferation was assessed with a Cell Counting Kit-8 (CCK-8, Dojindo, Japan). Cells were seeded into 96-well plates (5 × 10^3^/well) 48 h after transfection with si-TMEM106C or si-NC. Then, 10 μl of CCK8 solution was added per well 2 h before the end of incubation at 37° C every 12h. Absorbance (450 nm) was assessed with an ELISA reader (MuLTiSKAN MK3, Thermo, USA). The viability is given as a percent of the control value.

### *In vitro* cell migration and invasion assay

A total of 6×10^4^ transfected cells in FBS-free medium were seeded into the upper portion of transwell chambers (Matrigel-coated (BD, USA) or not) with an 8.0 μm pore membrane (Costa, Corning, NY. USA), while the lower compartment contained 10% FBS. After 48h, cells remaining on the upper surface were removed, while cells adhering to the chamber’s lower surface were fixed with 4% paraformaldehyde and then were stained using 0.05% crystal violet. Ten random fields of stained cells that migrated or invaded were assessed with an inverted microscope (Olympus, Tokyo, Japan).

### Western blot analysis and immunohistochemistry (IHC)

Tissue lysate (50 μg) was separated by SDS-polyacrylamide gel electrophoresis (SDS-PAGE) and then was transferred to nitrocellulose membranes (Whatman, UK). The membranes were incubated overnight with rabbit anti-human TMEM106C (UNIV-BIO, Shanghai, China) and mouse anti-human GAPDH (Cell Signaling Technology, USA) antibodies; then, they were incubated for 60-90mins with a horseradish peroxidase-conjugated secondary antibody (goat-anti-rabbit IgG, LI-COR, USA). Then, membranes were scanned by the Odyssey infrared imaging system (LI-COR, USA). IHC analysis was performed with TMEM106C antibodies using methods and as described previously [55].

### GEPIA and oncomine analysis

The mRNA expression of TMEM106C in HCC was first analyzed by the GEPIA (Gene Expression Profiling Interactive Analysis) database (gepia.cancer-pku.cn) based on TCGA (The Cancer Genome Atlas) and GTex (The Genotype-Tissue Expression) databases, including 369 tumor specimens and 160 normal liver specimens. Then, the expression level of TMEM106C in HCC was further investigated within the Oncomine 4.5 database (https://www.oncomine.org). The Oncomine^TM^ platform contains 715 gene expression data sets and data from 86,733 cancer tissues and normal tissues [20]. The analyses were generated from the following series of HCC-related studies: Chen Liver (N = 75, T = 104), Mas Liver (N = 19, T = 38), Roessler Liver (N = 21, T = 22), Roessler Liver 2 (N = 220, T = 225) and Wurmbach Liver (N = 10, T = 35) studies [56–59]. *P* < 0.01 was considered significant.

### UALCAN analysis

UALCAN (http://ualcan.path.uab.edu), a database that is easy to use, is an interactive web portal to perform in-depth analyses of TCGA gene expression data. UALCAN uses TCGA level 3 RNA-seq and clinical data from 31 cancer types. This portal’s user-friendly features allowed us to analyze the relative expression of a query gene(s) across tumor and normal samples, as well as in various tumor subgroups based on individual cancer stages, tumor grade, race, body weight or other clinicopathologic features. This database serves as a platform for *in silico* validation of target genes and for identifying tumor subgroup-specific candidate biomarkers [60]. Here, the expression level of TMEM106C across tumor and normal samples was again analyzed in UALCAN. Additionally, the relative expression relations between TMEM106C and clinicopathological features such as sex, age, race, cancer stage and tumor grade were analyzed.

### LinkedOmics analysis

The LinkedOmics database contains multiomics data and clinical data for 32 cancer types and a total of 11158 patients from TCGA project [45]. We used the LinkFinder module to investigate genes that were differentially expressed in correlation with TMEM106C based on TCGA liver hepatocellular carcinoma (LIHC) data (N = 371). The results are presented in volcano plots and heat maps, which were analyzed statistically using Pearson’s correlation coefficient. Then, the GSEA tool (Gene Set Enrichment Analysis) in the LinkInterpreter module was used to perform Go and KEGG pathway analysis of differentially expressed genes, as well as kinase-target enrichment, miRNA-target enrichment and transcription factor-target enrichment. The rank criterion was FDR < 0.05, and 500 simulations were performed. The results are presented in bar charts and tables. LinkedOmics is openly available at http://www.linkedomics.org.

### GeneMANIA analysis

GeneMANIA (http://www.genemania.org) is a flexible, user-friendly web interface that enables users to construct a composite gene-gene functional interaction network from a gene list. The resulting network includes genes that are most related to the original list and functional annotations, which is generated by Gene Ontolog [61]. We selected the significant genes associated with the biological function of TMEM106C, which were identified as being enriched in HCC by GSEA: CDK1 kinase gene set (CDC25C, RFC4, KIF2C, BIRC5, and CDC20), transcription factor E2F1 gene set (E2F1, CDT1, CDC6, EZH2, and MCM2) and miR-19A/miR-19B gene set (DLC1, FBXO8, TGFBR2, SH3D19, and NRBF2). The items in GeneMANIA are defined as follows: coexpression, physical interaction, colocalization, predicted and pathway. The predicted functions based on a large database of functional interaction networks from multiple organisms are also listed. Two genes are linked if their expression levels are similar based on a gene expression study.

### Statistical analysis

For biological validation data, values are presented as the mean ± SD. Student’s t test was used to determine significance. All statistical tests were two-sided. Data were imaged with GraphPad Prism 7.0 software. Within LinkedOmics analysis, a Pearson correlation test was employed to analyze the correlation between TMEM106C and the other genes within the LinkedOmics analysis. The false discovery rate (FDR) method was used to adjust the *P* value for multiple hypothesis testing. FDR <0.05 was established as the threshold [62, 63]. *P* < 0.05 was considered statistically significant.

## Supplementary Material

Supplementary Figure 1

Supplementary Table 1

Supplementary Tables 2, 3 and 4
