# Supplementary material for: TMEM106C contributes to the malignant characteristics and poor prognosis of hepatocellular carcinoma
Source: Aging (Albany NY). 2021 Feb 11;13(4):5585–606. doi: 10.18632/aging.202487 (PMC7950261; doi:10.18632/aging.202487)
Supplement: Supplementary Figure 1 [file aging-13-202487-s001.pdf]

SUPPLEMENTARY FIGURE

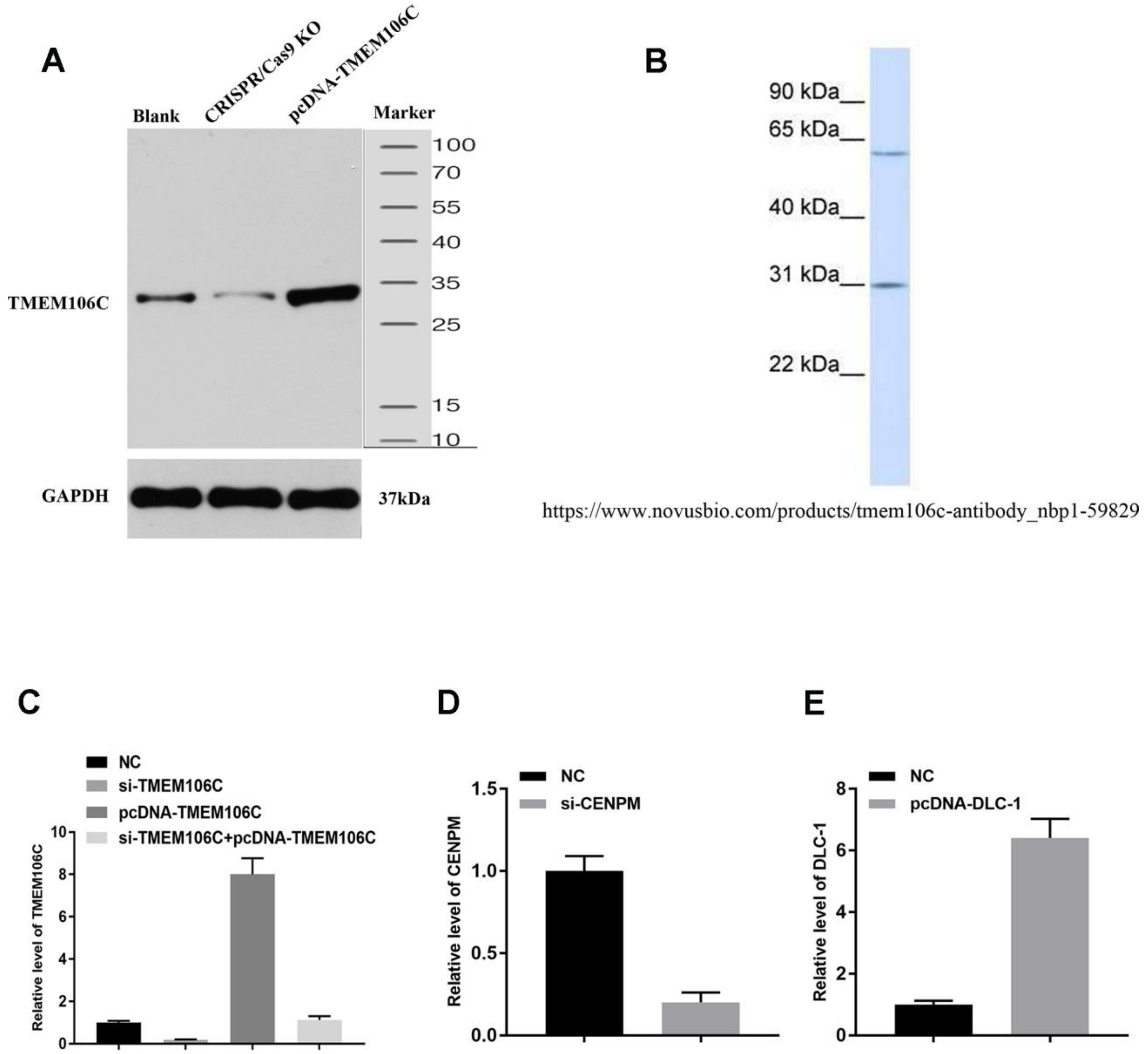

Supplementary Figure 1. (**A**) WB of TMEM106C in 7721 cells transfected with a TMEM106C CRISPR/Cas9 KO plasmid or a pcDNA-TMEM106C expression plasmid. (**B**) The WB image following detection by TMEM106C antibody from the Novus website. (**C**) Relative levels of TMEM106C in 7721 cells transfected with si-TMEM106C or a pcDNA-TMEM106C expression plasmid. (**D**) Relative level of CENPM in 7721 cells transfected with si-CENPM. (**E**) Relative level of DLC-1 in 7721 cells transfected with a pcDNA-DLC-1 expression plasmid.
