# Supplementary material for: TMEM106C contributes to the malignant characteristics and poor prognosis of hepatocellular carcinoma
Source: Aging (Albany NY). 2021 Feb 11;13(4):5585–606. doi: 10.18632/aging.202487 (PMC7950261; doi:10.18632/aging.202487)
Supplement: Supplementary Tables 2, 3 and 4 [file aging-13-202487-s003.pdf]

SUPPLEMENTARY TABLES

Supplementary Table 2. Sequences of primers for human genes analyzed by real-time PCR.

Real-time PCR prime	Sense (5'→3')	Antisense (5'→3')
TMEM106C	CGAAGAGGCTGGACATGACA	GGCTTCTTCCTGCTCTCGTT
GAPDH	CATCATCCCTGCCTCTACTGG	CTGGTGAATTTTACCGGGAAG
DLC-1	TCCTCTCCATCAGGCACACC	TCCGTGGACTCAGTGTCAGAAG
CENPM	CACGGAGGATGCTCTTCTGC	CACGATCAGGTCAATTCGGG

Supplementary Table 3. Sequences of the inserted fragment in pcDNA-TMEM106C and pcDNA-DLC1 plasmids.

TMEM106C ORF	ATGGGGTCTCAGCATTCCGCTGCTGCTCGCCCCTCCTCCTGCAGGCGAAAGCAAGAAGATGA<br>CAGGGACGGTTTGCTGGCTGAACGAGAGCAGGAAGAAGCCATTGCTCAGTTCCCATATGTGG<br>AATTCACCGGGAGAGATAGCATCACCTGTCTCACGTGCCAGGGGACAGGCTACATTCCAACA<br>GAGCAAGTAAATGAGTTGGTGGCTTTGATCCCACACAGTGATCAGAGATTGCGCCCTCAGCG<br>AACTAAGCAATATGTCCTCCTGTCCATCCTGCTTTGTCTCCTGGCATCTGGTTTGGTGGTTTTC<br>TTCCTGTTTCCGCATTCAGTCCTTGTGGATGATGACGGCATCAAAGTGGTGAAAGTCACATTT<br>AATAAGCAAGACTCCCTTGTAATTCTCACCATCATGGCCACCCTGAAAATCAGGAACTCCAAC<br>TTCTACACGGTGGCAGTGACCAGCCTGTCCAGCCAGATTCAGTACATGAACACAGTGGTGAA<br>TTTTACCGGGAAGGCCGAGATGGGAGGACCGTTTTCCTATGTGTACTTCTTCTGCACGGTACC<br>TGAGATCCTGGTGCACAACATAGTGATCTTCATGCGAACTTCAGTGAAGATTTCATACATTGGC<br>CTCATGACCCAGAGCTCCTTGGAGACACATCACTATGTGGATTGTGGAGGAAATTCCACAGCT<br>ATTTAA
DLC1 ORF	ATGAAGCTAGAAATTAGTCCTCATCGGAAACGAAGTGACGATTCAGACGAGGATGAGCCTTG<br>TGCCATCAGTGGCAAATGGACTTTCCAAAGGGACAGCAAGAGGTGGTCCCGGCTTGAAGAG<br>TTTGATGTCTTTTCTCCAAAACAAGACCTGGTCCCTGGGTCCCCAGACGACTCCCACCCGAA<br>GGACGGCCCCAGCCCCGGAGGCACGCTGATGGACCTCAGCGAGCGCCAGGAGGTGTCTTCC<br>GTCCGCAGCCTCAGCAGCACTGGCAGCCTCCCCAGCCACGCGCCCCCCAGCGAGGATGCTGC<br>CACCCCCCGGACTAACTCCGTCATCAGCGTTTGCTCCTCCAGCAACTTGGCAGGCAATGACG<br>ACTCTTTCGGCAGCCTGCCCTCTCCCAAGGAACTGTCCAGCTTCAGCTTCAGCATGAAAGGC<br>CACGAAAAAACTGCCAAGTCCAAGACGCGCAGTCTGCTGAAACGGATGGAGAGCCTGAAGC<br>TCAAGAGCTCCCATCACAGCAAGCACAAAGCGCCCTCAAAGCTGGGGTTGATCATCAGCGGG<br>CCCATCTTGCAAGAGGGGATGGATGAGGAGAAGCTGAAGCAGCTCAACTGCGTGGAGATCTC<br>CGCCCTCAATGGCAACCGCATCAACGTCCCCATGGTACGAAAGAGGAGCGTTTCCAACTCCA<br>CGCAGACCAGCAGCAGCAGCAGCCAGTCGGAGACCAGCAGCGCGGTCAGCACGCCCAGCCC<br>TGTTACGAGGACCCGGAGCCTCAGTGCGTGCAACAAGCGGGTGGGCATGTACTTAGAGGGCT<br>TCGATCCTTTCAATCAGTCAACATTTAACAACGTGGTGGAGCAGAACTTTAAGAACCGCGAG<br>AGCTACCCAGAGGACACGGTGTTCTACATCCCTGAAGATCACAAGCCTGGCACTTTCCCCAA<br>AGCTCTCACCAATGGCAGTTTCTCCCCCTCGGGGAATAACGGCTCTGTGAACTGGAGGACGG<br>GAAGCTTCCACGGCCCTGGCCACATCAGCCTCAGGAGGGAAAACAGTAGCGACAGCCCCAA<br>GGAACTGAAGAGACGCAATTCTTCCAGCTCCATGAGCAGCCGCCTGAGCATCTACGACAACG<br>TGCCGGGCTCCATCCTCTACTCCAGTTCAGGGGACCTGGCGGATCTGGAGAACGAGGACATC<br>TTCCCCGAGCTGGACGACATCCTCTACCACGTGAAGGGGATGCAGCGGATAGTCAATCAGTG<br>GTCGGAGAAGTTTTCTGATGAGGGAGATTCGGACTCAGCCCTGGACTCGGTCTCTCCCTGCC<br>CGTCCTCTCCAAAACAGATACACCTGGATGTGGACAACGACCGAACCACACCCAGCGACCTG<br>GACAGCACAGGCAACTCCCTGAATGAACCGGAAGAGCCCTCCGAGATCCCGGAAAGAAGGG<br>ATTCTGGGGTTGGGGCTTCCCTAACCAGGTCCAACAGGCACCGACTGAGATGGCACAGTTTC<br>CAGAGCTCACATCGGCCAAGCCTCAACTCTGTATCACTACAGATTAACTGCCAGTCTGTGGCC<br>CAGATGAACCTGCTGCAGAAATACTCACTCCTAAAGCTAACGGCCCTGCTGGAGAAATACAC<br>ACCTTCTAACAAGCATGGTTTTAGCTGGGCCGTGCCCAAGTTCATGAAGAGGATCAAGGTTCC<br>AGACTACAAGGACCGGAGTGTGTTTGGGGTCCCACTGACGGTCAACGTGCAGCGCACAGGA<br>CAACCGTTGCCTCAGAGCATCCAGCAGGCCATGCGATACCTCCGGAACCATTGTTTGGATCAG<br>GTTGGGCTCTTCAGAAAATCGGGGGTCAAGTCCCGGATTCAGGCTCTGCGCCAGATGAATGA<br>AGGTGCCATAGACTGTGTCAACTACGAAGGACAGTCTGCTTATGACGTGGCAGACATGCTGA<br>AGCAGTATTTTCGAGATCTTCCTGAGCCACTAATGACGAACAAACTCTCGGAAACCTTTCTAC<br>AGATCTACCAATATGTGCCCAAGGACCAGCGCCTGCAGGCCATCAAGGCTGCCATCATGCTGC<br>TGCCTGACGAGAACCGGGAGGTTCTGCAGACCCTGCTTTATTTCCTGAGCGATGTCACAGCA<br>GCCGTAAAAGAAAACCAGATGACCCCAACCAACCTGGCCGTGTGCTTAGCGCCTTCCCTCTT<br>CCATCTCAACACCCTGAAGAGAGAGAATTCCTCTCCCAGGGTAATGCAAAGAAAACAAAGTT<br>TGGGCAAACCAGATCAGAAAGATTTGAATGAAAACCTAGCTGCCACTCAAGGGCTGGCCCAT<br>ATGATCGCCGAGTGCAAGAAGCTTTTCCAGGTTCCCGAGGAAATGAGCCGATGTCGTAATTCC<br>TATACCGAACAAGAGCTGAAGCCCCTCACTCTGGAAGCACTCGGGCACCTGGGTAATGATGA<br>CTCAGCTGACTACCAACACTTCCTCCAGGACTGTGTGGATGGCCTGTTTAAAGAAGTCAAAG<br>AGAAGTTTAAAGGCTGGGTCAGCTACTCCACTTCGGAGCAGGCTGAGCTGTCCTATAAGAAG<br>GTGAGCGAAGGACCCCCTCTGAGGCTTTGGAGGTCAGTCATTGAAGTCCCTGCTGTGCCAGA<br>GGAAATCTTAAAGCGCCTACTTAAAGAACAGCACCTCTGGGATGTAGACCTGTTGGATTCAAA<br>AGTGATCGAAATTCTGGACAGCCAAACTGAAATTTACCAGTATGTCCAAAACAGTATGGCACC<br>TCATCCTGCTCGAGACTACGTTGTTTTAAGAACCTGGAGGACTAATTTACCCAAAGGAGCCTG<br>TGCCCTTTTACTAACCTCTGTGGATCACGATCGCGCACCTGTGGTGGGTGTGAGGGTTAATGT<br>GCTCTTGTCCAGGTATTTGATTGAACCCTGTGGGCCAGGAAAATCCAAACTCACCTACATGTG<br>CAGAGTTGACTTAAGGGGCCACATGCCAGAATGGTACACAAAATCTTTTGGACATTTGTGTGC<br>AGCTGAAGTTGTAAAGATCCGGGATTCCTTCAGTAACCAGAACACTGAAACCAAAGACACCA<br>AATCTAGGTGA

Supplementary Table 4. siRNA interference sequences of TMEM106C and CENPM.

TMEM106C	Start	Sequence
	891	GAGAGCACAGCAUAUGUUC^{dTdT}
CENPM	Start	Sequence
	607	GGAUGUCCAGUCAGGGCUU^{dTdT}
